# Influence of induced electric field on cold brew coffee: Temperature rise, physicochemical properties, and shelf life

**DOI:** 10.1016/j.fochx.2024.102036

**Published:** 2024-11-22

**Authors:** Yuhang Wu, Na Yang, Zhenlei Xiao, Yangchao Luo, Yamei Jin, Man Meng, Xueming Xu

**Affiliations:** a School of Food Science and Technology, Jiangnan University, 1800 Lihu Road, Wuxi 214122, China; b Department of Nutritional Sciences, University of Connecticut, Storrs, CT 06269, United States; c Licheng Detection & Certification Group Co., Ltd., 6 Shennong Road, Zhongshan 528437, China

**Keywords:** Flavor characteristics, Induced electric field, Microbial inactivation, Pasteurization, Storage

## Abstract

Cold brew coffee has gained significant popularity in the global market. This study examined the differences in chemical properties and flavor of cold brew coffee during storage, which was subjected to low-temperature pasteurization using induced electric field (IEF) at temperatures of 52 °C and 58 °C for 92 s, corresponding to 18.52 V/cm and 25.92 V/cm. Then, a high-temperature short-time (HTST) pasteurization was performed at 93 °C for 2 min as the control. Microbial analysis demonstrated that IEF treatment at 58 °C achieved a bactericidal effect. Both the IEF and HTST groups exhibited consistent trends in total sugar and total phenol content, showing approximately 28 μg GAE/mL after 28 days for IEF-2 group, compared to 25 μg/mL for HTST. Flavor analysis indicated that IEF group preserved the aroma characteristics during storage period. Further, IEF treatment effectively retained the key aroma compounds in cold brew coffee through GC–MS analysis, particularly pyrazine compounds with a relative content increased by 0.96 % in IEF-2 group after 28 days. Moreover, the bioactive compounds initially increased and subsequently decreased over the storage.

## Highlights

• Cold brew coffee was pasteurized using induced electric field at 52 °C and 58 °C.
• Increased voltage and induced current raised terminal temperature of the coffee.
• Induced electric field inactivated *Staphylococcus aureus* and *Escherichia coli* at 58 °C.
• Induced electric field treatment effectively retained key aroma compounds.
• Induced electric field treatment minimizes flavor loss during the storage.

## 1 Introduction

As an integral part of coffee consumption, cold brew coffee is becoming increasingly popular among people due to its unique taste and flavor. The production involves slowly steeping the ground coffee for several hours at a low temperature, maximizing the retention of aroma compounds, while reducing the bitterness and acidity. Therefore, the treatment of cold brew coffee at low-temperature offers significant advantages of preserving its flavor and quality over storage periods, which is crucial for the coffee industry because it meets consumer demand for a high-quality coffee drink as well as ensures a consistent product quality throughout supply chain (Pramudya & Seo, 2018).

Currently, conventional methods for treating coffee beverages include ultra-high temperature (UHT) treatment and other thermal pasteurization (Liu et al., 2022). Although thermal treatments are essential for eliminating microorganisms from the brewed coffee, they adversely affect its flavor and taste by destructing the key aroma components (Kumazawa & Masuda, 2003). Moreover, lots of aroma and flavor compounds in cold brew coffee are highly heat-sensitive; therefore, improper heat processing degrades these compounds, reducing consumer acceptance and impacting economic value (Guerra-Garcia et al., 2019).

Induced electric field (IEF) technology is a novel electric field processing method, which shows promises for heat-sensitive beverages. Different from conventional heating, the IEF treats liquid foods both through the thermal effects of induced currents and the non-thermal effects of magneto-induced electric field, thereby preserving its original flavor and nutritional components at mild temperature. For instance, the IEF was effective to kill microorganisms in apple juice at relatively low temperatures without affecting its nutrients (Wu et al., 2020). Similarly, IEF treatment was found to extend the shelf life of kiwi juice (He et al., 2021). These literature suggested that the IEF technology was effective for treating acidic liquid foods. But there is no reports on the IEF processing of cold brew coffee as well as the influence of flavor components during the storage.

Therefore, this study investigated the influence of IEF treatment on cold brew coffee to evaluate its feasibility as a potential processing method at low temperature of 52 °C and 58 °C for 92 s to see whether it preserved the key flavor of cold brew coffee at moderate field strength, while achieving microbial safety and storage stability. Then, the experiments were conducted to assess pH level, soluble solids content, conductivity, titratable acidity, total sugar content, and total phenol content after the treatments. Moreover, microbiological analysis included the pasteurization of *Staphylococcus aureus* and *Escherichia coli*. During storage for 28 days, the flavor characteristics was analyzed using electronic nose, gas chromatography–mass spectrometry (GC–MS), and high-performance liquid chromatography (HPLC).

## 2 Materials and methods

Cold brew coffee was obtained from White Dwarf Company (Wuxi, China). To prepare the sample, roasted coffee beans were ground to fine powder. Then, coffee was extracted by mixing the powder with distilled water at a ratio of 1:15 by keeping it for 10 h at around 5 °C. Once the extraction was complete, the mixture was cooled rapidly, which was then filtered and centrifuged to remove impurities; the supernatant liquid was the coffee extract. Standards for caffeine, chlorogenic acid, and trigonelline were provided by Meidesheng Technology Co., Ltd. (Chengdu, China). Folin-Ciocalteui phenol reagent was purchased from InnoChem Science & Technology Co., Ltd. (Beijing, China). Phenol (AR), 98 % concentrated sulfuric acid (AR), phosphoric acid (AR), methanol (SR), and all other chemical reagents were of analytical grade, which were supplied by Sinopharm Chemical Reagent Co., Ltd. (Shanghai, China).

### 2.1 Treatment procedure

**Fig. 1:**
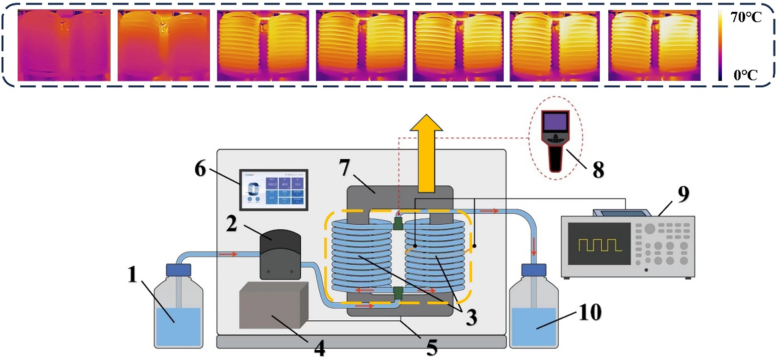
Instrumental chain contains the following components: 1. Inlet bottle, 2. Peristaltic pump, 3. Coil pipeline, 4. Power source, 5. Excitation coil, 6. Control panel, 7. Magnetic core, 8. Thermal imager, 9. Oscilloscope, and 10. Outlet bottle.

A continuous-flow IEF apparatus (MP10, Biomag Innovations, LCC, USA) used in this experiment was the same principle in previous literature (Yang et al., 2024) for the processing of cold brew coffee (Fig. 1). It generated an oscillating magnetic field around the magnetic circuit at an intermediate frequency through an excitation coil. Cold brew coffee was pumped through a spiral pipeline with an inner diameter of 6.4 mm, achieving electromagnetic coupling, thereby an IEF and induced current loaded on the flowing coffee.

The coffee sample was transported with a flow rate of 8.5 L/h at an initial temperature 25 ± 1 °C with the retention time in pipeline of 92 s. When the excitation voltage was set to 500 and 700 V at a frequency of 50 kHz, the IEF strength (DSO-X 2012 A, Agilent Technologies Inc., Palo Alto, USA) recorded by the oscilloscope was 18.52 and 25.92 V/cm, respectively. An infrared thermometer (H10, Hangzhou Hikvision Digital Technology Co., Ltd., China) was located at the distance of 15 cm to the outlet to record the real-time temperature of samples. The treated coffee was rapidly adjusted to 20 °C through a cooling pipeline and packaged through an aseptic filling machine, which were labeled, respectively as IEF-1 and IEF-2 groups. The control group was treated at 92 °C for 2 mins and labeled as the high-temperature short-time (HTST) group. After the treatments, the samples were stored in a refrigerator at 5 °C for 28 days. Subsequent analyses were conducted on the 7th, 14th, 21st and 28th days for each group.

### 2.2 Determination of pH, TSS, and conductivity

The pH, total soluble solids (TSS), and conductivity of samples were measured following the method of Cruz-O'Byrne et al. (2020) with minor modifications. The pH and TSS values were measured using a digital pH meter (Starter 3100, Ohaus Instrument Co., Ltd., China) and a portable refractometer (WZS-20, Shanghai Yidian Physical Optical Instrument Co., Ltd., China), respectively. The pH meter and conductivity meter were calibrated prior to use, while the refractometer was reset before measuring TSS. All instruments were cleaned between measurements to ensure the accuracy.

### 2.3 Microbial analysis

Following the method of Griffin et al. (2024) with minor modifications, *S. aureus* CMCC26003 and *E. coli* ATCC25922 were chosen as the representative pathogens for microbial analysis. The strains were gradually thawed from frozen tubes and transferred to nutrient agar plates for maintenance. Before using them, the strains were cultured in nutrient broth at 37 °C for 12 h with shaking at 200 rpm so as to get its logarithmic growth phase. Bacterial cells were collected after centrifuging at 8,000 rpm and 4 °C for 15 mins; after discarding the supernatant, the cell residue was resuspended in distilled water and centrifuged to remove soluble ions. Such washing process was repeated three times. The concentration of bacterial suspension was adjusted to 10^7^ to 10^8^ CFU/mL and added to the coffee solution at a concentration of 1 %, setting the initial bacterial concentration of 10^5^ to 10^6^ CFU/mL. Microbial count analysis was conducted after inoculation and incubation. Results were expressed in terms of log CFU/mL.

### 2.4 Titratable acidity

Titratable acidity (TA) was determined using the method of Basheer et al. (2022) with minor modification. Briefly, 5 mL of cold brew coffee was accurately weighed in 50-mL conical flask and recorded the mass as M. The titration was performed using 0.1 mol/L NaOH standard solution, monitoring with a pH meter until it reaches 7. The volume of NaOH solution was recorded as V. Total acidity calculated as chlorogenic acid was determined using the formula:

$$\text{Total Acidity Yield}\;\left(\%\right)=\frac{C\times V\times K}{M}\times 100$$

where, C is the concentration of NaOH solution (mol/L); V is the volume of NaOH solution (mL); K is the conversion factor for the acid (0.059 for chlorogenic acid); and M is the mass of cold brew coffee sample (g).

### 2.5 Determination of total sugar content

Following the method of Yue et al. (Yue et al., 2022) with minor modification, total sugar content was determined. Cold brew coffee (2 mL) was taken in a 25 mL colorimetric tube, which was mixed with 1 mL of 5 % phenol solution (prepared by mixing 1 mL of 80 % phenol solution with 15 g of distilled water) and 5 mL of 98 % concentrated sulfuric acid, gently rotating the tube during addition to ensure thorough mixing. Record the time immediately after adding sulfuric acid and after 10 min, the tube was placed in a room temperature water-bath for 5 min to cool. Then, the absorbance was measured at 490 nm, using distilled water as a blank for the calibration. Total sugar content was determined by comparing the sample's absorbance to glucose standard curve, expressed as the weight of glucose.

### 2.6 Determination of total phenolic content

Following the method of Alnsour et al. (2022) with minor modification, total phenolic content was determined by transferring 0.1 mL of sample to a 10 mL volumetric flask in which 6 mL of distilled water and 0.5 mL of Folin-Ciocalteu reagent were added and mixed well. Within 0.5 to 8 min, 1.5 mL of 20 % Na_2_CO_3_ solution was added and then diluted to the mark with distilled water. Let the mixture stand at room temperature for 2 h before measuring the absorbance at 765 nm with a reagent blank as the reference. Calculate phenol concentration of each sample according to gallic acid calibration curve, which was then expressed as the weight of gallic acid.

### 2.7 Electronic nose analysis

The aroma profile of samples was analyzed using an electronic nose (Heracles 2, Alpha MOS, France), following the method of Aunsa-Ard and Kerdcharoen (2022) with an appropriate modification. The sample volume was 5 mL, in a 20 mL vial. The equilibration process was conducted at 60 °C for 25 min, with shaking at 500 rpm. A 100 μL aliquot was injected at a temperature of 250 °C, at a rate of 125 μL/s. The carrier gas flow rate was maintained at 10 mL/min. Analytes were collected over a temperature range of 50 °C to 240 °C, with a final collection temperature of 240 °C. The column temperature program involved an initial ramp of 1 °C/s to 80 °C, followed by a ramp of 2 °C/s to 250 °C, with a 60-s hold at the final temperature. Detection was performed using a flame ionization detector (FID) at 260 °C, with a detection time of 177 s. The FID amplification factor was set to 12. Calibration was conducted using nC2 – nC16 normal alkane standards, with retention indices calculated. Qualitative analysis of each compound was performed using the AroChemBase database.

### 2.8 GC–MS analysis

Volatile compounds in the samples were analyzed using a GC–MS system (GCMS-QP2020 NX, Shimadzu, Kyoto, Japan), following the method of Dong et al. (2019) with minor modification. Briefly, 5 g liquid sample was incubated at 60 °C for 40 mins and a CAR/DVB/PDMS triple-phase extraction head was used for the extraction. After adsorption, the extraction head was immediately inserted into the injection port for desorption, which lasted 10 mins. The extraction head was then cleaned at 250 °C for 3 mins.

GC conditions: A DB-WAX polar column (30 m × 0.25 mm; df = 0.25 μm; Agilent 122–7062, CA, USA) was used with total analysis time of 46.3 mins. The injection temperature was set to 250 °C and the program was as follows: Initial temperature was 35 °C (held for 10 mins), increased to 172 °C at a rate of 5 °C/min, and then to 230 °C at a rate of 15 °C/min (held for 5 mins). Injection was performed in a splitless mode.

MS conditions: The mass spectrometer operated in electron ionization (EI) mode was used with an electron energy of 70 eV. The scanning range was set from 50 *m*/*z* to 350 m/z, while both ion source and interface temperatures were maintained at 250 °C.

Qualitative analysis: Volatile components were preliminarily identified by comparing the detected spectra with the NIST Mass Spectral Library (NIST17), supplemented by relevant literature (Phillips et al., 2019).

### 2.9 HPLC analysis

HPLC analysis was conducted following the method of Atlabachew et al. (2021) with minor modification. For sample preparation, accurately measured 1 mL sample was diluted ten-folds with 0.1 % phosphoric acid solution. The mixture was then centrifuged at 10,000 rpm for 15 mins. The supernatant was filtered through a 0.45 μm aqueous filter membrane and stored at 4 °C until analysis.

HPLC analysis was performed using a Waters 1525EF system (Waters Corporation, Milford, MA, USA). The separation was achieved on an Xbridge C18 chromatographic column (4.6 mm × 250 mm, 5 μm) at a column temperature of 30 °C. The mobile phase consisted of methanol and 0.1 % phosphoric acid in water, with a flow rate of 0.5 mL/min and an injection volume of 10 μL. The UV wavelengths were set at 245 nm for trigonelline, 280 nm for caffeine, and 325 nm for chlorogenic acid. The gradient elution was performed as outlined in Table S1.

### 2.10 Statistical analysis

All experiments were conducted in triplicate and the results were expressed as mean ± standard deviation. Data were analyzed using one-way analysis of variance (ANOVA) with SPSS 26.0 software (IBM, USA). The *P* value of <0.05 was considered statistically significant.

## 3 Results and discussion

### 3.1 Temperature rise characteristics

As shown in Table S2, when the input voltage was increased, the amplitudes of IEF and induced current rose significantly. For instance, with an input voltage of 500 V, IEF strength (*E*) was 18.52 V/cm and induced current (*I*_*rms*_) was 0.62 A. When the input voltage was increased to 700 V, *E* and *I*_*rms*_ increased to 25.92 V/cm and 0.90 A, respectively, indicating a positive correlation between input voltage as well as both IEF strength and its current intensity. These findings highlighted the influence of electric field strength and temperature on the heating behavior of cold brew coffee. As the voltage increased, more magnetic energy was converted to thermal energy, thus accelerating the temperature rise. The heat generated was also proportional to induced current density applied on the sample, based on Joule's law. Interestingly, IEF treatment ensured a uniform distribution of heat, resulting a consistent increase in the sample temperature, which improved the process efficiency (Zhang et al., 2022).

**Fig. 2:**
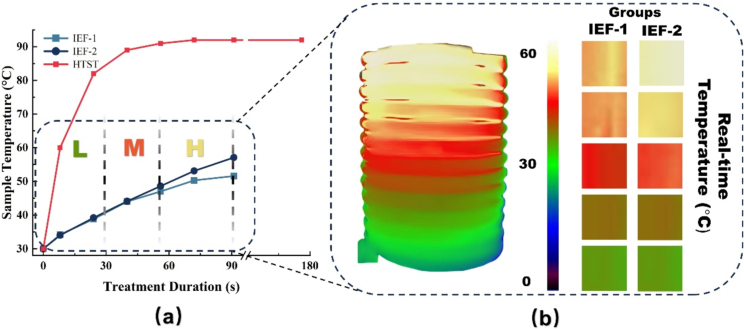
Temperature profiles and thermal imaging of cold brew coffee during IEF treatment. (a) Temperature rise curves; and (b) Thermal imaging during heating process under different conditions.

Fig. 2 illustrates the temperature rise curves for IEF-1 and IEF-2 groups, along with thermal images during the process. Meanwhile, the infrared temperature image in Fig. 1 depicts the temperature distribution of the secondary coil during the entire heating process. As shown in Fig. 2a, the terminal temperature for IEF-1 group was 52 °C, while the IEF-2 group reached 58 °C. This difference resulted from the strength of electric field (He et al., 2021; Wu et al., 2020). The duration to terminal temperature in the IEF groups was influenced by process factors, including field strength, current intensity, and fluid flow rate (Wu et al., 2022).

As shown in the thermal images in Fig. 2b, the temperature distribution during IEF-1 and IEF-2 processes was categorized into three zones, namely high-temperature, medium-temperature, and low-temperature. The low-temperature zone for both groups ranged from 30 to 40 °C, with a heating period of 32 s. Meanwhile, the medium-temperature zone ranged from 40 to 47 °C for IEF-1, while from 40 to 49 °C for IEF-2, with a heating period of 23 s. During the process, the medium and low-temperature zones were located in the middle and lower area of coil pipeline, maintaining consistent heating rates. It was caused by the lower initial temperature of cold brew coffee, which created a significant temperature gradient, enhancing thermal conduction efficiency (Wilhelmy et al., 2021). The high-temperature zone ranged from 47 to 52 °C for IEF-1, while from 49 to 60 °C for IEF-2, with a heating period of 37 s. In this temperature zone, the upward trend of current density slowed down, while heat loss increased, slowing the temperature rise. The IEF process ensured uniform temperature distribution within liquid foods under the action of oscillating magnetic field, at the beginning stage, achieving a linear temperature increase (He et al., 2021). It might prevent local overheating because of smaller pipeline cross-sectional area. Uniform heating process protected heat-sensitive components, leading to optimal inactivation (Kumazawa & Masuda, 2003; Yıkmış, 2016).

### 3.2 Physicochemical properties

Table 1 presents the changes in pH, TSS, and conductivity of cold brew coffee during storage. At the beginning, there were minimal differences in pH, TSS, and conductivity among the groups. However, pH level gradually decreased with storage time, compared to other indicators, it shown the most significant decline. The reduction likely resulted from the production of acidic metabolic products initially present before the treatment, which effectively reduced microbial activity. Meanwhile, the decrease of activity limited further production of acidic compounds, stabilizing its pH at a lower level (Wang et al., 2021). Industrial production systems also showed a decrease in pH value during long-term storage (HTST treatment decreases by 0.35 after 270 days of storage) (Maksimowski et al., 2023). The TSS values exhibited slight fluctuations during the period, with no noticeable differences observed in its external color (Fig. S1). This aligns with the findings by Li et al. (2021) in case of the non-thermal treatment of cantaloupe juice. Additionally, the conductivity of cold brew coffer remained around 15.4 mS/cm, with minimal variation between the treatments, suggesting that the conductivity was relatively stable during 28-days storage.

**Table 1:** Physicochemical properties of cold brew coffee during the storage.

Storage (d)	Parameters	Control Group	IEF-1 Group	IEF-2 Group	HTST Group
0	pH	5.51 ± 0.01^b^	5.46 ± 0.01^a^	5.50 ± 0.06^ab^	5.36 ± 0.07^a^
	TSS(°Brix)	12.1 ± 0.10^b^	12.3 ± 0.10^a^	11.7 ± 0.10^c^	12.3 ± 0.10^a^
	Conductivity (mS/cm)	15.6 ± 0.10^a^	15.6 ± 0.10^a^	15.6 ± 0.03^a^	15.6 ± 0.10^a^
7	pH	5.02 ± 0.02^a^	4.99 ± 0.02^ab^	4.95 ± 0.05^b^	4.97 ± 0.02^ab^
	TSS(°Brix)	12.2 ± 0.10^a^	12.1 ± 0.00^a^	11.6 ± 0.12^b^	12.1 ± 0.10^a^
	Conductivity (mS/cm)	15.0 ± 0.10^a^	15.1 ± 0.00^a^	15.1 ± 0.13^a^	15.1 ± 0.10^a^
14	pH	4.93 ± 0.01^ab^	4.91 ± 0.03^b^	4.78 ± 0.01^c^	4.95 ± 0.02^a^
	TSS(°Brix)	11.9 ± 0.00^a^	11.9 ± 0.00^a^	11.4 ± 0.15^b^	11.9 ± 0.00^a^
	Conductivity (mS/cm)	15.5 ± 0.10^a^	15.4 ± 0.10^a^	15.4 ± 0.06^a^	15.5 ± 0.00^a^
21	pH	4.91 ± 0.02^a^	4.93 ± 0.02^a^	4.68 ± 0.01^b^	4.93 ± 0.01^a^
	TSS(°Brix)	11.9 ± 0.10^a^	12.0 ± 0.10^a^	11.2 ± 0.00^b^	12.1 ± 0.20^a^
	Conductivity (mS/cm)	15.5 ± 0.00^a^	15.6 ± 0.10^a^	15.5 ± 0.11^a^	15.5 ± 0.10^a^
28	pH	4.88 ± 0.01^bc^	4.94 ± 0.03^a^	4.86 ± 0.06^b^	4.88 ± 0.01^bc^
	TSS(°Brix)	11.9 ± 0.00^b^	11.9 ± 0.00^b^	12.2 ± 0.06^a^	11.9 ± 0.10^b^
	Conductivity (mS/cm)	15.2 ± 0.00^c^	15.4 ± 0.00^b^	15.3 ± 0.06^c^	15.6 ± 0.10^a^

**Fig. 3:**
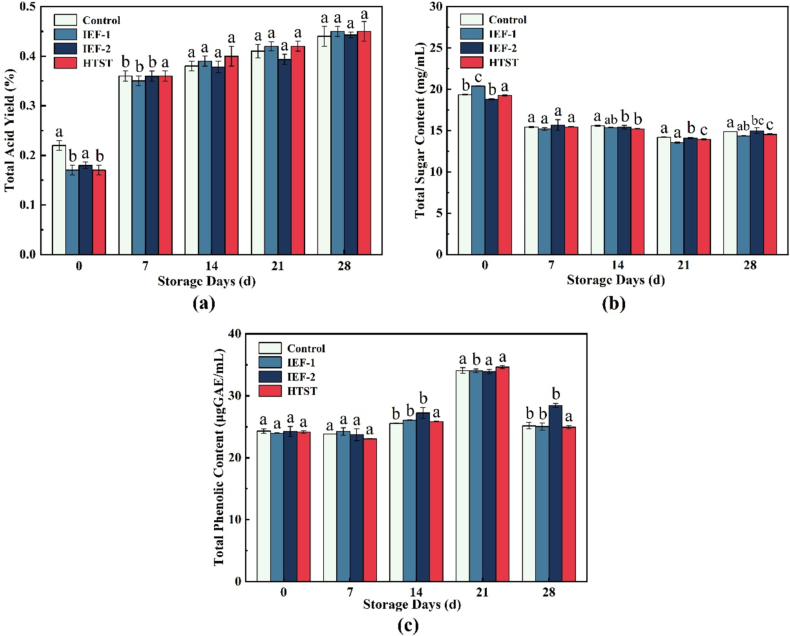
Changes in titratable acidity, total sugar, and total phenols of cold brew coffee during storage. (a) Titratable acidity; (b) Total sugar content; and (c) Total phenol content.

Fig. 3 illustrates the change trends of titratable acidity, total sugar, and total phenols in cold brew coffee during storage. Titratable acidity generally increased in all groups with storage time. Notably, the IEF-treated samples showed less fluctuation in titratable acidity than during HTST treatment, suggesting an advantage in preserving acidic compounds, which was beneficial to the quality of cold brew coffee. In this aspect, the electrical field treatment effectively preserved the acidity and flavor profile of beverages, demonstrating its superiority over high-temperature treatments (Zhu et al., 2019). Total sugar content remained unchanged between the IEFs and HTST groups, suggesting that both treatments had little impact on the sugar content of cold brew coffee.

At the early stage of storage (0 days), the IEF treated samples did not exhibit a significant advantage in preserving phenolic compounds, compared to other heat-treated samples. Despite the existence of non-thermal effect of IEF treatment, the phenolic content was comparable to that of HTST group, suggesting that the stability of these compounds was likely governed by multifaceted factors beyond the processing method alone.

Additionally, the IEF-2 group maintained total phenol content of approximately 28 μg GAE/mL after 28 days of storage, which was significantly higher than the HTST group (approximately 25 μg GAE/mL) and the IEF-1 group (approximately 25 μg GAE/mL). This observation was consistent with the findings by Ağçam, Akyıldız and Akdemir Evrendilek (2014), who concluded that the PEF treatment improved the stability of phenolic compounds in orange juice, with the retention of bioactive compounds, as influenced by the interplay of processing parameters and storage conditions. Meanwhile, the decrease in phenolic content as a result of HTST treatment of orange juice showed a consistent value (decreased by 25–30 %) (Ağçam and Akyıldız, 2014). In this case, the quality of cold brew coffee was also depended upon electric field strength. Ohmic heating at high electric field strengths enhanced the retention of total phenols due to the electroporation effect on cell membranes, which promoted the release and stability of phenolic compounds (Safarzadeh Markhali et al., 2022). Alternatively, the stability of phenolic compounds is closely related to the inhibition of enzyme activity, particularly polyphenol oxidase (PPO) and peroxidase (POD), which can degrade phenolic compounds during storage. Studies have shown that IEF can effectively inhibit these enzymes, contributing to the retention of phenolic compounds. For example, in kiwi juice, IEF treatment significantly inhibited PPO and POD activity through the combined effects of internal heating and enzyme denaturation, thereby improving the stability of phenolic compounds during storage (Zhang, Yang, et al., 2018).

### 3.3 Microbiological analysis

**Fig. 4:**
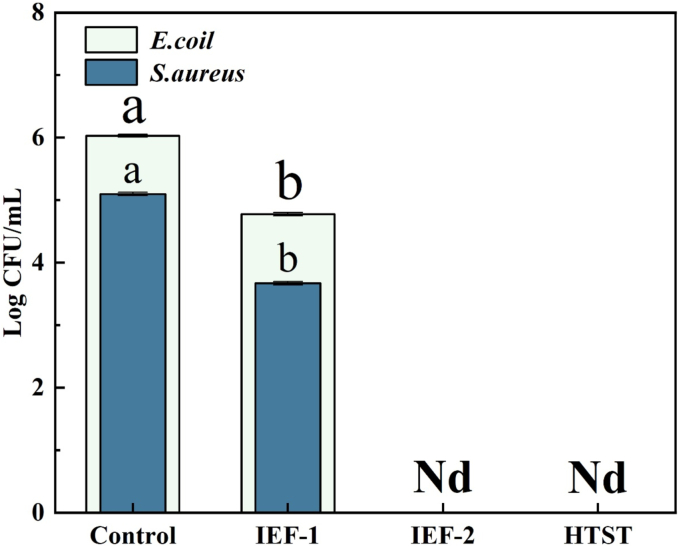
Inactivation effects of different treatments on cold brew coffee.

Fig. 4 illustrates the changes in colony forming unit of *S. aureus* and *E. coli* under different conditions. The initial counts of *S. aureus* and *E. coli* were approximately 6 log and 5 log, respectively. After the IEF treatment, bacterial counts in the samples were significantly reduced.

In the IEF-1 group, *S. aureus* decreased to 4.77 log, while *E. coli* decreased to 3.67 log. Although lower electric field strength and temperature conditions was able to reduce bacterial counts, initial inoculation levels might still result in residual bacteria (Bonetta et al., 2014). In contrast, the counts of *S. aureus* and *E. coli* decreased to undetectable levels in the IEF-2 group, indicating superior inactivation efficiency. It attributed not only to a higher terminal temperature in the IEF-2 group but also to the critical role of non-thermal effect. Thus, it enhanced electroporation on bacterial cell membranes, significantly reducing bacterial counts (Geveke & Brunkhorst, 2003). Furthermore, the electric field induced structural damage to the bilayer of cell membrane, further compromising microbial viability (Pillet et al., 2016).

After the HTST treatment, the counts of *S. aureus* and *E. coli* in cold brew coffee were also reduced to undetectable levels. The high-temperature treatment achieved the inactivation by denaturing as well as inactivating microbial proteins and enzymes, thereby disrupting cell structure and function, which ultimately led to microbial death (Kim et al., 2019). Comparatively, the IEF-1 group exhibited less inactivation than the HTST treatment, whereas the inactivation effect of IEF-2 group was comparable to that of HTST treatment. Overall, the IEF-2 group demonstrated optimal inactivation effect under moderate electric field strength and appropriate temperature, closely matching the efficacy of conventional heat treatment. Furthermore, the processing at lower temperatures may preserve the flavor and nutritional components of cold brew coffee, which is essential for maintaining its quality.

### 3.4 Electronic nose

**Fig. 5:**
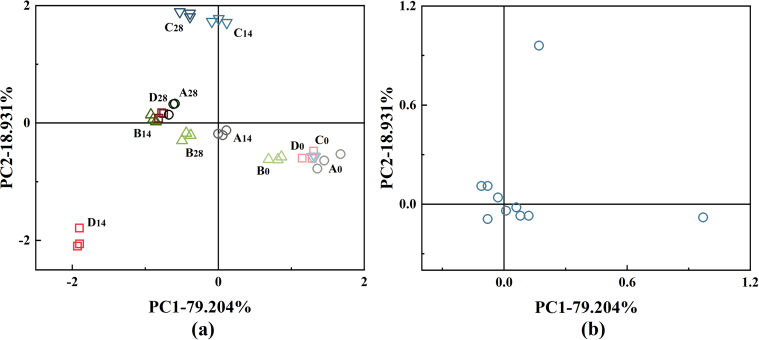
Electronic nose analysis of cold brew coffee after different treatments during storage. Score plot (a) and loading plot (b) derived from PCA analysis. The treatments include Control (A), IEF-1 (B), IEF-2 (C), and HTST (D). The numbers (0, 7, 14, 28) correspond to the storage periods in days.

Fig. 5 presents the electronic nose analysis of aroma characteristics in cold brew coffee under different treatments during storage. Fig. 5a revealed that principal component 1 (PC1) and principal component 2 (PC2), and highlighted key differences in the aroma characteristics with PC1 accounted for 79.20 % of the variance, while PC2 accounted for 18.93 %, making PC1 as the primary focus for the analysis. Fig. 5b presents the loading plot of chromatographic peaks. By screening peaks with significant sample differences (Discrimination Power > 0.900) and large peak areas (Range > 1000), good separation was achieved. The overall distribution trend of the samples, after peak screening, was consistent with the unscreened data in Fig. 5a, indicating that the selected contributing factors adequately reflect the overall odor profile of the sample group.

The clustering distribution along the PC1 axis for group A, B, C and D during storage showed that the IEF treatment affected the aroma properties of cold brew coffee. Notably, after 28 days of storage, the samples treated with IEF at 25.92 V/cm exhibited significant differences, compared to other groups. Increased electric field strength was likely to enhance the disruption of intermolecular interactions within the cold brew coffee, thereby facilitating the release of volatile aroma compounds. It also demonstrated that higher electric field intensities enhanced the release and production of volatile compounds, underscoring the efficacy of this approach in modulating aroma profiles (Delbaere et al., 2022).

In comparison, the HTST group showed less distinct volatile differences at the initial stages; however, these differences became more pronounced after 14 days of storage, suggesting that the cumulative effects of HTST treatment on aroma properties surpassed those of IEF treatment over time. This implied that the temperature-induced changes gradually became more evident as cold brew coffee interacted with its environment, leading to extensive alterations in volatile compounds, when compared to the IEF treatment.

Above results indicated that IEF treatment had certain advantages over HTST treatment in maintaining aroma characteristics of cold brew coffee. In this case, the IEF treatment preserved volatile aroma compounds while reducing negative effects, such as the loss of aroma and nutritional components due to temperature rise. It demonstrated that electronic nose could reliably distinguish coffee aroma variations after different processing conditions, highlighting the importance of optimal processing parameters to preserve coffee quality (Aunsa-Ard and Kerdcharoen, 2022).

### 3.5 SPME-GC–MS analysis

**Fig. 6:**
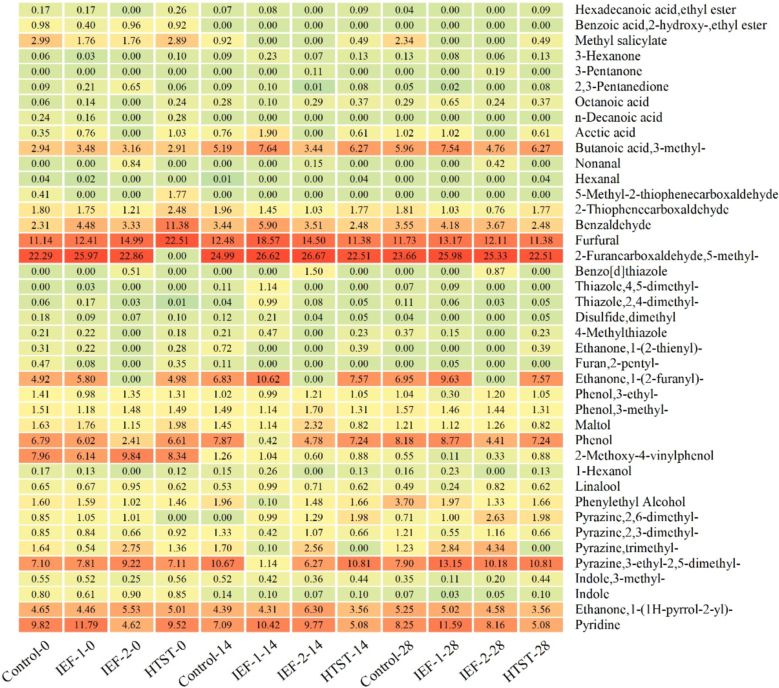
Changes in relative content of volatile compounds in cold brew coffee treated with IEF and HTST during storage. Note: Control-0, Control-14, and Control-28 represent untreated cold brew coffee stored for 0, 14, and 28 days, respectively. IEF-1-0, IEF-1-14, and IEF-1-28 represent cold brew coffee treated with IEF-1 stored for 0, 14, and 28 days, respectively. IEF-2-0, IEF-2-14, and IEF-2-28 represent cold brew coffee treated with IEF-2 stored for 0, 14, and 28 days, respectively. The redder the color, the higher the contents; the bluer the color, the lower the content. The contents in the table are all average values.

Fig. 6 shows the changes in the relative content of volatile compounds in cold brew coffee during storage after different treatments. Table S3 lists the 41 detected compounds, including pyridines, furans, ketones, aldehydes, phenols, pyrroles, pyrazines, esters, acids, and alcohols, along with their odor descriptions, corresponding to the compounds analyzed in Fig. 6.

Pyridine compounds, known for their unique sour, putrid, and fishy odors, were the focus of this section of analysis. At the beginning of storage, the relative content of pyridine in the IEF-1 group was at the highest level of 11.79 %. On the 28th day, the relative content of pyridine in the IEF-1 group decreased to 11.59 %. In comparison, the IEF-2 group, which reached a higher temperature of 58 °C, compared to the of temperature IEF-1 group (52 °C), showing a relatively lower pyridine content of 8.16 % on the 28th day, while the HTST group showed a further decrease to 6.27 %. It indicated that the HTST had a more pronounced effect on pyridine compounds than the IEF treatment. It also demonstrated that pyridine compounds were highly sensitive to roasting temperatures, leading to an increased pyridine content, which negatively influenced the overall aroma and flavor profile of coffee due to the unpleasant and bitter characteristics associated with them (Gancarz et al., 2022).

Compared to pyridines, pyrazine compounds (For example, 2-ethyl-3,6-dimethylpyrazine, contributing potato, cocoa, and roasted nut flavors) were of particular interest. The IEF treatment significantly increased pyrazine concentrations, with the IEF-1 group rising from 7.81 % (Day 0) to 13.15 % (Day 28), while the IEF-2 group rising from 9.22 % (Day 0) to 10.18 % (Day 28). These results suggested that the IEF treatment effectively enhanced pyrazine content, which was crucial for desirable roasted and nutty flavors in cold brew coffee, without causing the extensive degradation seen in conventional heat treatments. It demonstrated that roasting conditions, particularly storage temperature and time, played a critical role in the formation of pyrazines like key aroma compounds. Additionally, the literature highlighted that a precise control of these parameters was essential to achieve a desired aroma profile in coffee, as variations could influence the concentration and balance of these compounds, thereby improving the overall flavor of the cold brew coffee (Schenker et al., 2002).

Alcohols and phenols, key components for floral, woody, and complex flavors, also showed unique trends. The change in the content of 2-methoxy-4-vinylphenol was particularly noteworthy. After the storage, the content of 2-methoxy-4-vinylphenol significantly decreased in all groups; however, the fresh sample had a higher content of 0.55 %, compared to other groups. The degradation of volatile phenolic compounds was caused by a high temperature and oxidation (Zhang, Li, et al., 2018; Zhang, Yang, et al., 2018). Furthermore, increased electric field strengths have shown to influence flavor profile of beverages, by enhancing the retention and release of aroma compounds. The action leads to initial intensification of flavor, which remain stable during storage. The outcomes also have been well-described in studies examining the effects of PEF on liquid foods (Lasekan et al., 2017).

Notably, 5-methyl-2-furancarboxaldehyde and furfural were the compounds with highest relative contents, which are aldehyde compounds contributing to cold brew coffee flavor. The former is associated with caramel, cereal, and maple flavors. In HTST group, 5-methyl-2-furancarboxaldehyde was not detected, most probably because of high temperature and oxidation reactions, leading to the loss of this compound (Lolli et al., 2020). The furfural content was only at 0.05 % after the storage, likely because of the thermal degradation and breakdown of precursor compounds during the treatment. There was a significant decrease in furfural content during storage, most probably due to the decomposition into furans at a high temperature treatment (Scharf & Sandmann, 2023; Vasiliou et al., 2013). The IEF treatment was performed at lower temperatures, which effectively prevented the degradation of 5-methyl-2-furancarboxaldehyde and furfural, preserving these flavor compounds. Other volatile substances were recorded, which were not discussed in details here, because their changes were relatively minor.

### 3.6 HPLC analysis

As shown in Table 2, on the 0th day, caffeine content in the IEF-1 and IEF-2 group was 816.9 mg/L and 823.8 mg/L, increasing, respectively by 11.8 % and 12.7 %, when compared with untreated group. The IEF-2 group had 199.2 mg/L of chlorogenic acid, which was 14.8 % higher than that of the control. For trigonelline, the IEF-1 group had 236.1 mg/L on day 0, which was significantly higher than that of the control (210.6 mg/L). The initial increase in non-volatile compounds was likely due to the enhanced molecular interactions facilitated by the electric field, which promoted the release of these substances. The IEF treatment demonstrated a clear advantage in preserving these key compounds during the first 14 days of storage. However, by day 28, retention levels in the IEF groups were comparable to those in the HTST group, indicating a diminishing effect of IEF over extended periods.

**Table 2:** HPLC analysis of non-volatile compounds in cold brew coffee after different treatments during storage.

Detected Component (mg/L)	Storage Days (d)	Control Group	IEF-1 Group	IEF-2 Group	HTST Group
Caffeine	0	730.8 ± 4.13^c^	816.9 ± 3.67^a^	823.8 ± 3.93^a^	730.3 ± 1.69^b^
	14	857.7 ± 6.27^d^	927.4 ± 4.93^c^	962.6 ± 3.93^a^	948.0 ± 8.04^b^
	28	795.3 ± 0.44^c^	769.9 ± 1.18^d^	840.8 ± 1.71^b^	849.6 ± 1.51^a^
Chlorogenic Acid	0	173.5 ± 0.18^d^	192.7 ± 1.62^b^	199.2 ± 1.12^a^	187.4 ± 1.24^c^
	14	193.0 ± 0.27^c^	167.3 ± 1.32^d^	220.9 ± 1.73^a^	209.9 ± 2.31^b^
	28	174.4 ± 0.57^c^	168.5 ± 0.49^d^	180.5 ± 0.86^b^	183.9 ± 0.18^a^
Trigonelline	0	210.6 ± 0.48^d^	236.1 ± 1.14^a^	224.5 ± 0.99^c^	228.9 ± 1.58^b^
	14	243.6 ± 0.42^c^	213.5 ± 1.81^d^	250.1 ± 0.91^b^	266.8 ± 2.69^a^
	28	224.7 ± 0.81^b^	218.8 ± 0.49^d^	230.4 ± 1.11^c^	239.0 ± 0.13^a^

Caffeine, trigonelline, and chlorogenic acid are functional components in cold brew coffee. Among them, caffeine acts as a central nervous system stimulant, enhancing alertness and attention, also possesses antioxidant properties (Scharf & Sandmann, 2023). Trigonelline has anti-diabetic and anti-inflammatory properties, in addition to enhancing coffee flavor (Worku Wondimkun et al., 2022). Chlorogenic acid is a major antioxidant with anti-inflammatory, antibacterial, and blood pressure-lowering effects, contributing significantly to the health benefits of cold brew coffee (Bellumori et al., 2021).

## 4 Conclusion

From the inlet to the outlet of the pipeline, a significant temperature difference was observed in continuous-flow's cold brew coffee. Due to the inner heating effect as well as lower temperatures caused by appropriate IEF strength, the process could reduce the thermal damage to heat-sensitive components. Regarding microbial analysis, the IEF treatment demonstrated significant bactericidal effects on *S. aureus* and *E. coli* at 58 °C. It also effectively preserved sensory and nutritional quality of cold brew coffee, while achieving similar microbial inactivation as conventional pasteurization. In terms of physicochemical properties, all groups exhibited similar trends in pH, conductivity, and TSS values over the period. At higher field strengths, the IEF group retained certain flavor compounds, such as total phenols and pyrazines. Additionally, the IEF-2 group effectively increased the content of pyrazine compounds, which was crucial for forming desirable roasted and nutty flavors. Caffeine, chlorogenic acid, and trigonelline contents in IEF groups initially increased, followed by a decrease during the storage, suggesting that moderate IEF strength facilitated the release of key flavor compounds. In conclusion, the IEF technology offers opportunities for the processing of cold brew coffee at relatively low temperature. Through parallel pipeline design, processing capacity can be enlarged to large-scale level. Thus, the optimization of IEF parameters to enhance the flavor stability of cold brew coffee is necessary.

## CRediT authorship contribution statement

**Yuhang Wu:** Writing – review & editing, Writing – original draft, Validation, Methodology, Investigation, Formal analysis, Data curation, Conceptualization. **Na Yang:** Writing – review & editing, Resources, Project administration, Investigation, Funding acquisition. **Zhenlei Xiao:** Methodology, Conceptualization. **Yangchao Luo:** Methodology, Investigation. **Yamei Jin:** Supervision, Investigation, Funding acquisition. **Man Meng:** Validation. **Xueming Xu:** Supervision, Resources, Project administration, Investigation, Funding acquisition.

## Declaration of competing interest

The authors declare that they have no known competing financial interests or personal relationships that could have appeared to influence the work reported in this paper.

## Data Availability

Data will be made available on request.

## Appendix A Supplementary data

**Captions for all supplementary materials.** (e.g., **Fig. S1** Photos of cold brew coffee after different treatments. **Table S1** Gradient elution program. **Table S2** IEF and induced current at different input voltages of the treatments.) Image 1
