# Supplementary material for: Influence of induced electric field on cold brew coffee: Temperature rise, physicochemical properties, and shelf life
Source: Food Chem X. 2024 Nov 22;24:102036. doi: 10.1016/j.fochx.2024.102036 (PMC11647621; doi:10.1016/j.fochx.2024.102036)
Supplement: Supplementary file 1 — Captions for all supplementary materials. (e.g., Fig. S1 Photos of cold brew coffee after different treatments. Table S1 Gradient elution program. Table S2 IEF and induced current at different input voltages of the treatments.) [file mmc1.docx]

**Fig. S1.** Photos of cold brew coffee after different treatments.

**
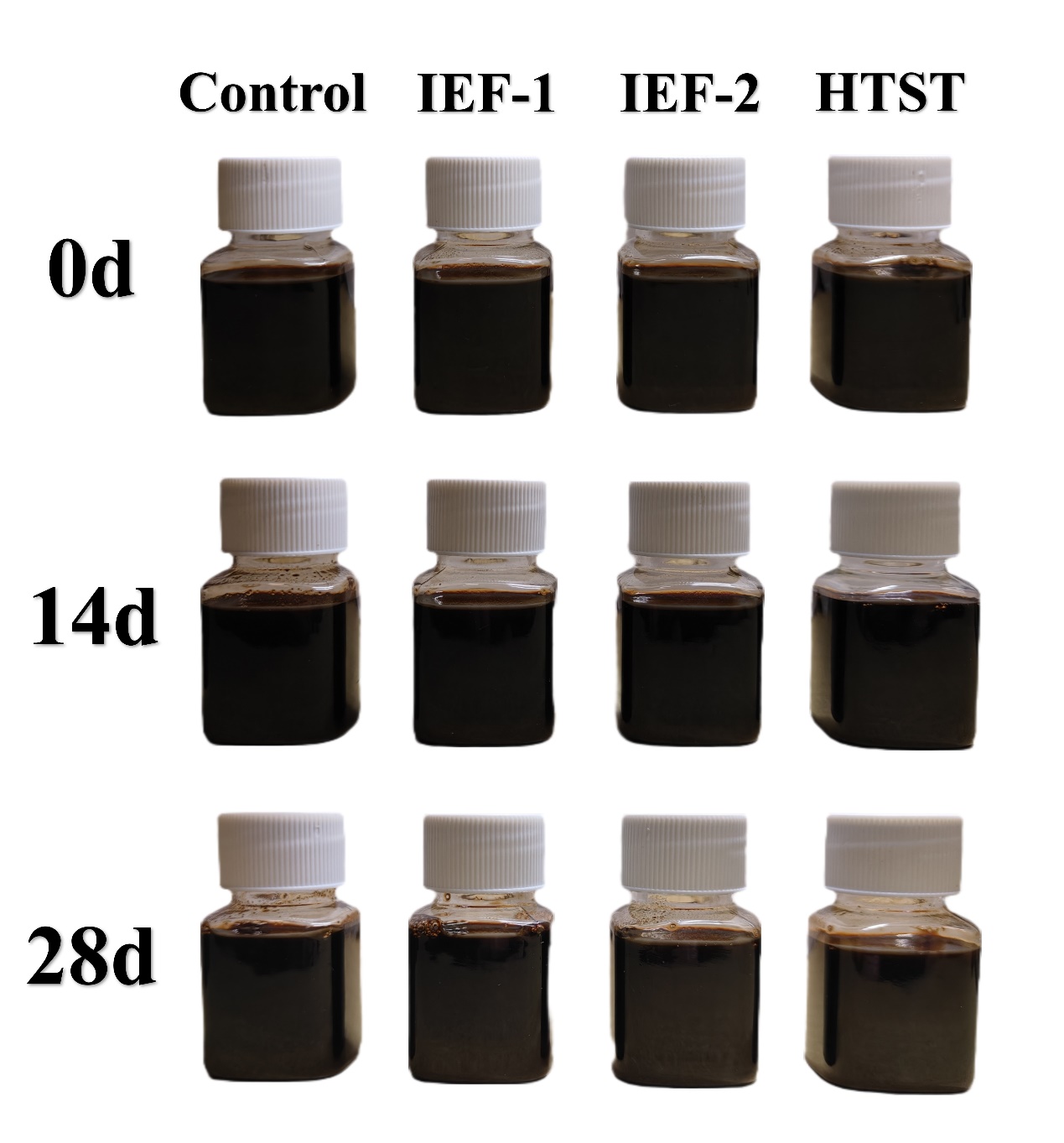
Figure S1**

**Table S1.** Gradient elution program.

| Time（min） | C（%） | D （%） | | Time（min） | C（%） | | D （%） |
| --- | --- | --- | --- | --- | --- | --- | --- |
| 0 | 0 | 100 | 45 | | | 100 | 0 |
| 5 | 0 | 100 | 50 | | | 0 | 100 |
| 30 | 50 | 50 | 60 | | | 0 | 100 |
| 40 | 100 | 0 |  | | |  |  |

**Table S2.** IEF and induced current at different input voltages of the treatments.

| Input voltage (V) | Induced voltage (*U*) (V) | Induced current (*I_rms_*) (A) | IEF strength (*E*) (V/cm) |
| --- | --- | --- | --- |
| 500.00 | 235.00 | 0.62 | 18.52 |
| 700.00 | 330.00 | 0.90 | 25.92 |

**Table S3.** Identified volatile compounds in cold brew coffee across different treatments.

| No. | Category | Compound name | Odor description |
| --- | --- | --- | --- |
|  |  |  |  |
|  |  |  |  |
| 1 | Pyridines | Pyridine | Sour, putrid, fishy |
| 2 |  | Ethanone, 1-(1H-pyrrol-2-yl)- | Musty nut shell, licorice, walnut, bread |
| 3 |  | Indole | Animal floral, mothball |
| 4 |  | Indole, 3-methyl- | Civet excrement |
| 5 | Pyrazines | Pyrazine, 3-ethyl-2,5-dimethyl- | Potato, cocoa, roasted nuts |
| 6 |  | Pyrazine, trimethyl- | Nutty, earthy, cocoa, roasted potato, hazelnut, musty |
| 7 |  | Pyrazine, 2,3-dimethyl- | Nutty peanut butter, coffee, walnut, caramel, roasted |
| 8 |  | Pyrazine, 2,6-dimethyl- | Cocoa, roasted nuts, roasted beef, coffee |
| 9 | Alcohols | Phenylethyl Alcohol | Floral rose, dry rose, rose water |
| 10 |  | Linalool | Citrus floral, sweet rose, woody, blueberry |
| 11 |  | 1-Hexanol | Green grassy, fruity, apple peel |
| 12 | Phenols | 2-Methoxy-4-vinylphenol | Dry woody, fresh amber, roasted peanut |
| 13 |  | Phenol | Plastic, rubbery |
| 14 |  | Maltol | Caramel, cotton candy, toasted bread |
| 15 |  | Phenol, 3-methyl- | Medicinal woody, leather |
| 16 |  | Phenol, 3-ethyl- | Musty |
| 17 | Furans | Ethanone, 1-(2-furanyl)- | Sweet balsamic, almond, cocoa, caramel |
| 18 |  | Furan, 2-pentyl- | Fruity, earthy, vegetal, metallic |
| 19 | Sulfur Compounds | Ethanone, 1-(2-thienyl)- | Nutty, hazelnut, walnut |
| 20 |  | 4-Methylthiazole | Nutty, tomato |
| 21 |  | Disulfide, dimethyl | Cabbage, onion |
| 22 |  | Thiazole, 2,4-dimethyl- | Coffee, tea, beefy |
| 23 |  | Thiazole, 4,5-dimethyl- | Roasted nut, fishy |
| 24 |  | Benzo[d]thiazole | Gasoline, rubber |
| 25 | Aldehydes | 2-Furancarboxaldehyde, 5-methyl- | Caramel, cereal, maple |
| 26 |  | Furfural | Sweet woody, almond, toasted bread |
| 27 |  | Benzaldehyde | Almond, cherry |
| 28 |  | 2-Thiophenecarboxaldehyde | Sulfurous |
| 29 |  | 5-Methyl-2-thiophenecarboxaldehyde | Sweet almond, cherry, woody |
| 30 |  | Hexanal | Green grassy, fruity |
| 31 |  | Nonanal | Fatty, citrusy |
| 32 | Acids | Butanoic acid, 3-methyl- | Sour, tropical cheese |
| 33 |  | Acetic acid | Vinegar |
| 34 |  | n-Decanoic acid | Rancid fatty citrusy |
| 35 |  | Octanoic acid | Rancid oily, cheesy |
| 36 | Ketones | 2,3-Pentanedione | Roasted, buttery, caramel |
| 37 |  | 3-Pentanone | Ether |
| 38 |  | 3-Hexanone | Sweet fruity, waxy rum, grape |
| 39 | Esters | Methyl salicylate | Wintergreen minty |
| 40 |  | Benzoic acid, 2-hydroxy-, ethyl ester | Minty floral, spicy |
| 41 |  | Hexadecanoic acid, ethyl ester | Fruity creamy, balsamic |

Note: Odor description from http://www.flavornet.org.
